# A mild thermomechanical process for the enzymatic conversion of radiata pine into fermentable sugars and lignin

**DOI:** 10.1186/s13068-017-0748-6

**Published:** 2017-03-09

**Authors:** Ian D. Suckling, Michael W. Jack, John A. Lloyd, Karl D. Murton, Roger H. Newman, Trevor R. Stuthridge, Kirk M. Torr, Alankar A. Vaidya

**Affiliations:** 1 Scion, 49 Sala St, Rotorua, 3046 New Zealand; 2 Department of Physics, University of Otago, PO Box 56, Dunedin, 9054 New Zealand; 3 FP Innovations, 2665 East Mall, Vancouver, BC V6T 1Z4 Canada

**Keywords:** Pine, Ball-milling, Biofuels, Softwood, Enzymatic conversion, Galactoglucomannans, Sugar yield, Energy

## Abstract

**Background:**

Conversion of softwoods into sustainable fuels and chemicals is important for parts of the world where softwoods are the dominant forest species. While they have high theoretical sugar yields, softwoods are amongst the most recalcitrant feedstocks for enzymatic processes, typically requiring both more severe pretreatment conditions and higher enzyme doses than needed for other lignocellulosic feedstocks. Although a number of processes have been proposed for converting softwoods into sugars suitable for fuel and chemical production, there is still a need for a high-yielding, industrially scalable and cost-effective conversion route.

**Results:**

We summarise work leading to the development of an efficient process for the enzymatic conversion of radiata pine (*Pinus radiata*) into wood sugars. The process involves initial pressurised steaming of wood chips under relatively mild conditions (173 °C for 3–72 min) without added acid catalyst. The steamed chips then pass through a compression screw to squeeze out a pressate rich in solubilised hemicelluloses. The pressed chips are disc-refined and wet ball-milled to produce a substrate which is rapidly saccharified using commercially available enzyme cocktails. Adding 0.1% polyethylene glycol during saccharification was found to be particularly effective with these substrates, reducing enzyme usage to acceptable levels, e.g. 5 FPU/g OD substrate. The pressate is separately hydrolysed using acid, providing additional hemicellulose-derived sugars, for an overall sugar yield of 535 kg/ODT chips (76% of theoretical). The total pretreatment energy input is comparable to other processes, with the additional energy for attrition being balanced by a lower thermal energy requirement. This pretreatment strategy produces substrates with low levels of fermentation inhibitors, so the glucose-rich mainline and pressate syrups can be fermented to ethanol without detoxification. The lignin from the process remains comparatively unmodified, as evident from the level of retained β-ether interunit linkages, providing an opportunity for conversion into saleable co-products.

**Conclusions:**

This process is an efficient route for the enzymatic conversion of radiata pine, and potentially other softwoods, into a sugar syrup suitable for conversion into fuels and chemicals. Furthermore, the process uses standard equipment that is largely proven at commercial scale, de-risking process scale-up.

## Background

Advanced biofuels derived from lignocellulosic biomass, composed of cellulose, hemicellulose and lignin, are seen as a key to the future growth of biofuels. They are not derived from food crops and promise to be more sustainable, offering greater reductions in greenhouse gas emissions compared to conventional biofuels [1]. Potential lignocellulosic feedstocks include wood and wood residues, agricultural residues such as corn stover or sugarcane bagasse and dedicated energy crops such as miscanthus or energy cane.

One of the most promising approaches to the production of lignocellulosic biofuels involves using enzymes to hydrolyse the carbohydrate polymers in the substrate to monomeric sugars and then fermenting these sugars to ethanol [2]. Critical to high sugar yields during enzymatic hydrolysis is an effective pretreatment to disrupt and/or remove the lignin and hemicelluloses encasing the cellulose microfibrils and make the cellulose more accessible to the enzymes [3–9].

Heating lignocellulosic biomass in water, or directly with steam, is one of the simplest and most effective pretreatments. There are many variants on this basic approach, with most hydrothermal pretreatments involving heating the biomass to temperatures of between 160 and 230 °C, often in the presence of acid catalysts [3–5, 10]. However, hydrothermal pretreatments suffer from a number of disadvantages. Firstly, under the acid conditions, the hemicelluloses may be hydrolysed and degraded to produce furans and acetic acid, which inhibit subsequent fermentation stages [11], and into pseudo-lignin, which can deposit on cellulose surfaces and retard enzymatic hydrolysis [12]. Furthermore, the lignin in the biomass can be modified in the pretreatment process to produce compounds which inhibit subsequent saccharification or fermentation [11, 13, 14] and lignin can also be relocalised within the cell wall to negatively impact cellulose hydrolysis [15–17].

Mechanical milling processes such as ball-milling can also be used to improve the enzymatic hydrolysis of lignocellulosic materials by increasing the surface area of the cellulose. However, the amount of energy required is normally considered to be prohibitively high [18]. Nevertheless, a number of researchers have suggested that refining or ball-milling at moderate energy inputs can be beneficially applied to increase digestibility after hydrothermal and chemical pretreatments [19–25].

Softwoods, such as *Pinus radiata*, pose particular challenges in enzymatic processes. Firstly, softwoods are amongst the most recalcitrant lignocellulosic substrates in enzymatic processes, typically requiring both more severe pretreatment conditions and higher enzyme doses than hardwoods or agricultural residues [6, 26, 27]. Secondly, galactoglucomannans (GGMs) are the dominant hemicellulose sugars in softwoods, whereas xylans are the main hemicelluloses in hardwoods and agricultural residues [28]. With GGMs making up 15–20% of the wood mass in softwoods, efficient conversion of this polymer to its constituent C6 sugars is critical for good overall yields. While a number of pretreatments have been investigated for softwoods [6], including processes based on steam explosion [29–31], single- and two-stage acid treatments [32, 33], sulphite treatments [34, 35], organosolv pulping [36] and alkaline pulping [20, 37], there is still a need for a high-yielding, industrially scalable and cost-effective pretreatment for these substrates.

We describe here a new efficient process for the conversion of softwoods into monomeric sugars in high yields. This process, developed in a programme of work, involves a novel combination of known steps combined and operated in a specific way, affording high yields of fermentable monomeric sugars using reasonable doses of current commercial enzyme cocktails. Specifically here we describe the overall process, including its rationale and overall performance, with recent [38, 39] and future publications providing more detail on the specific steps within the process.

## Results and discussion

### Process overview

**Fig. 1:**
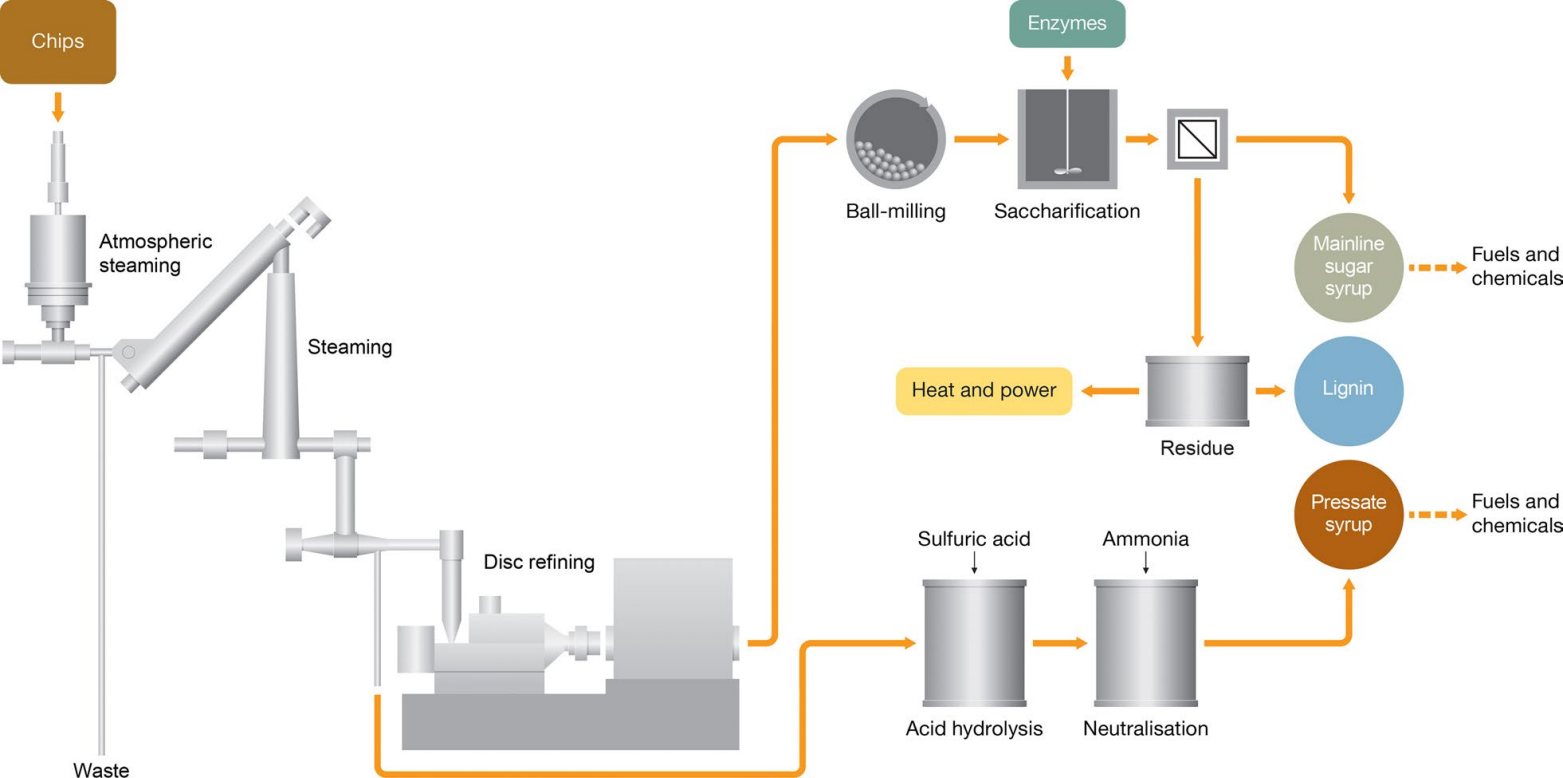
Process overview

Our process is illustrated in Fig. 1. It involves a two-stage pretreatment, composed of a mild thermomechanical stage without added acid catalyst, combined with mechanical attrition using a ball-mill. The thermomechanical stage can be carried out in equipment similar to that used commercially for the production of fibre for medium density fibreboard and produces a solid residue largely as individual fibres. While the fibres themselves are not responsive to enzymatic hydrolysis, attrition by wet ball-milling yields a digestible substrate. Treatment of this ball-milled substrate with commercially available enzyme cocktails affords a mainline sugar syrup containing the monomeric sugars, plus a solid residue containing mainly lignin and unhydrolysed carbohydrates. The solid residue can either be used to isolate the lignin for downstream processing, or be burnt as source of process energy.

To increase the overall monomeric sugar yields, a compression screw is incorporated between the steaming and disc refining stages to squeeze out a pressate rich in solubilised hemicelluloses. The resulting pressate is hydrolysed with dilute acid in a subsequent step to afford a pressate syrup containing mainly C6 hemicellulose-derived monomeric sugars. In this way, the GGMs can be converted to the constituent monomeric sugars without requiring an enzyme cocktail containing the enzymes needed to fully degrade these hemicelluloses.

Each of these steps is then discussed in more detail in subsequent sections, illustrated with data from two steaming conditions.

### Thermomechanical pretreatment

Thermomechanical pretreatment was carried out at a pilot scale in equipment commonly used during the production of wood fibre for newsprint and medium density fibreboard [40]. Fresh *P. radiata* chips were first softened by atmospheric steaming at 80 °C for 5 min and then passed through a compression screw. This squeezed out a small amount of material, ca. 0.5% on OD chips, which was discarded as waste, as it contains low levels of sugars. It is however rich in wood extractives, which could potentially be isolated as a saleable co-product.

The compressed chips were then steamed at 7.5 bar (173 ± 2 °C) and passed through a second compression screw to afford a concentrated pressate rich in hemicellulose sugars, plus a solid residue. We have recently reported that when the steaming time at 173 °C is increased from 3 to 144 min, a greater proportion of the hemicelluloses are solubilised and removed into the pressate [38]. Notably, these steaming conditions are mild relative to those commonly employed during other dilute acid treatments and steam explosion treatments. For example, steaming at 173 °C for 72 min corresponds to combined severity factor [41] of 0.57 versus 1.4–5.4 for steam pretreatment of softwoods in the presence of added acid catalysts [42].

The solid residue was disc-refined under pressure to produce a pulp containing largely individual fibres. Under these conditions, the fibres separate at the lignin-rich middle lamella layer, as the temperature exceeds the glass transition temperature of lignin [43]. The nominal refining energy here is 300 kWh/ODT. However, refining energies during similar commercial processes, e.g. medium density fibreboard production, are considerably lower than required in our pilot plant due to the larger scale and optimised plate design, typically ~120 kWh/ODT (0.43 GJ/ODT) [44].

Table 1 shows that the fibre yield decreases as the steaming time is increased from 3 to 72 min. This is principally due to greater hydrolysis and subsequent dissolution of hemicelluloses, as evident from the drop in mannan and xylan content of the fibres [38]. Little cellulose is solubilised.

**Table 1:** Mass and component balances for trials using 3- and 72-min steaming

	Initial wood^a^	3-min steaming^b^	72-min steaming^c^
Combined severity factor		−1.16	0.57
Wood or fibre, kg/ODT wood			
Mass	1000	949 (10)	838 (16)
Extractives	8 (3)	7 (2)	15 (3)
Lignin	290 (15)	272 (15)	278 (8)
Carbohydrates			
Arabinosyl	13 (2)	7 (2)	1 (1)
Galactosyl	23 (3)	19 (2)	7 (1)
Glucosyl	441 (13)	440 (18)	439 (15)
Xylosyl	50 (4)	45 (3)	30 (2)
Mannosyl	113 (3)	98 (6)	45 (3)
Pressate			
Concentration, g/L		58 (5)	127 (24)
Mass, kg/ODT wood		40 (5)	151 (10)
Total carbohydrates, kg/ODT wood		24 (3)	107 (11)
Arabinosyl		4 (1)	5 (1)
Galactosyl		3 (0)	13 (2)
Glucosyl		4 (1)	19 (2)
Xylosyl		3 (1)	12 (1)
Mannosyl		11 (1)	58 (6)
Ball-milled fibre digestibility^d^, % of glucosyl residues		57 (4)	73 (2)
Monomeric sugars, kg/ODT initial wood^e^		356 (24)	501 (14)
Mainline		327 (24)	388 (17)
Pressate		29 (5)	114 (9)

Average (standard deviation)

^a^ *n* = 8 batches of chips

^b^ *n* = 5 for pilot plant trials, *n* = 3 for digestibility

^c^ *n* = 8 for pilot plant trials, *n* = 6 for digestibility

^d^Fibre vibratory ball-milled for 60 min and digestibility determined using Celluclast 1.5L (20 FPU/OD g substrate) supplemented with β-glucosidase (Novozyme 188, 25 CBU/OD g substrate) for 24 h

^e^Monomeric sugar yields (as free sugars) from the pressates calculated from the pressate composition assuming the conversion efficiency shown in Fig. 3 and for the mainline syrup using the data in Fig. 7 to estimate the conversion of the other monomeric sugars from the yields of glucosyl residues to monomeric sugars

**Fig. 2:**
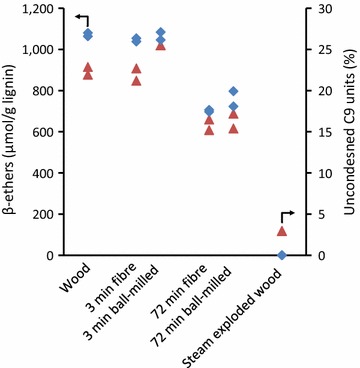
Levels of β-*O*-4 ether linkages (*diamonds*) and uncondensed phenylpropane units in lignins (*triangles*) from untreated and pretreated substrates. Results of duplicate determinations shown

The lignin residue remaining after the enzyme-based process for converting lignocellulosic substrates to sugars is a potential future feedstock for conversion into a wide variety of sustainable products [45, 46]. Under the acidic conditions present during hydrothermal treatment lignin can be both partially depolymerised via cleavage of β-*O*-4 ether interunit linkages and repolymerised via condensation between C-α and the aromatic ring of adjacent phenylpropane units [47–49]. We therefore investigated the extent to which the lignin was being modified in our process. Analysis of the levels of β-*O*-4 ether interunit linkages in the pretreated substrates by thioacidolysis [50] showed that the level of β-ethers remained essentially unchanged in the fibre after steaming for 3 min, and decreased by only ~30% after 72-min steaming (Fig. 2). In contrast, the lignin in a reference steam-exploded wood (SEW) prepared from *P. radiata* wood using the conditions identified for this substrate by Clark and Mackie [29] contained no detectable β-*O*-4 ethers. Furthermore, analysis of the lignin by nitrobenzene oxidation [51] showed that uncondensed phenylpropane units decreased by ~30% after 72-min steaming, compared to a >80% reduction in the reference SEW substrate. These results show that, relative to the reference steam explosion pretreatment, the lignins from this process have undergone only limited modification during steaming.

### Hemicellulose-rich pressate

The pressate from steaming for 72 min contained high concentrations, averaging 127 g/L, of hemicellulose sugars, making it particularly suitable for subsequent processing (Table 1). This is because steaming is carried out at high solids loading using direct steam heating, so only a low amount of pressate is produced, ca. 1.3 kg/kg OD chips entering the process.

The hemicellulose sugars are largely present in the pressate as soluble oligomers, with the concentrations increasing as the steaming time is increased (Table 1). In the pressate after 72-min steaming, the galactan:glucan:mannan ratio was 0.7:1:3.0, consistent with removal of GGMs from the wood and little cellulose dissolution. Softwoods are believed to contain two different GGMs, the galactan-rich having a galactan:glucan:mannan ratio of 1:1:3 and the other having a ratio of 0.1–0.2:1:3–4 [28], so our results suggest preferential dissolution of the galactan-rich GGM. Smaller amounts of arabinoxylans were also removed, but in this case largely as the monomeric sugars.

For pressates produced from 72-min steaming, levels of the fermentation inhibitors acetic acid (2.5–4.4 g/L) and furans (1.5–3.3 g/L furfural + hydroxymethylfurfural) are sufficiently low that detoxification is not required prior to fermentation (see below). By comparison, the liquid from the reference SEW treatment contained 7.1 g/L of acetic acid and 4.0 g/L of furfural plus hydroxymethylfurfural.

**Fig. 3:**
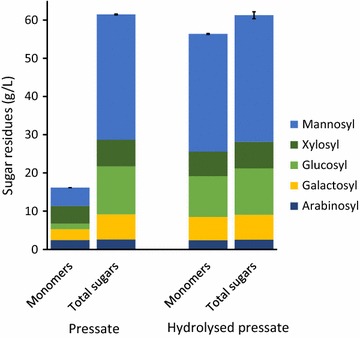
Sugar residues in pressate from one trial where *P. radiata* chips were steamed for 72 min, before and after hydrolysis at 121 °C in the presence of 1% sulphuric acid. Total sugars = sum of monomeric plus oligomeric sugars

Hydrolysis of the soluble oligomers in the pressate to the constituent monomers, required for many end-use applications such as fermentation to ethanol, can be accomplished by heating the pressate at 121 °C for 60 min in the presence of 1% w/w sulphuric acid (Fig. 3). More severe hydrolysis conditions could be used to ensure hydrolysis of the 8% remaining oligomers. However, this must be balanced against the greater pentose sugar degradation under these conditions [52] and higher costs due to higher temperatures, longer times and additional chemicals for hydrolysis and neutralisation.

### Ball-milling

**Fig. 4:**
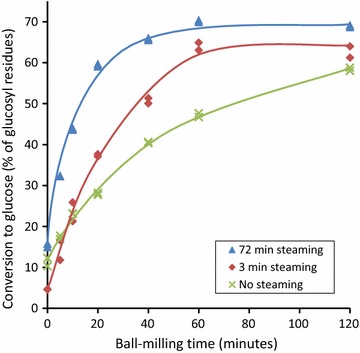
Impact of vibratory ball-milling on digestibility. Digestibility determined using Celluclast 1.5L (20 FPU/OD g substrate) supplemented with β-glucosidase (Novozyme 188, 25 CBU/OD g substrate). Data not corrected for ash produced during ball-milling

Wet ball-milling in a vibratory mill at 5% solids content dramatically increased the digestibility of the steamed fibre substrates [38]. Figure 4 shows that both the responsiveness of the fibre to ball-milling and the eventual extent of conversion to glucose after ball-milling for 120 min decreased in the order of 72-min steaming >3-min steaming >no steaming. The greater responsiveness of the more severely pretreated fibres to ball-milling is consistent with earlier results which have found that treatments which remove the hemicelluloses and/or lignin weaken the network structure of the polymer matrix, reducing the energy requirement for mechanical attrition [22, 24, 53–55]. In a closely aligned study, Shikinaka et al. [56] very recently reported that softwoods can be converted into glucose in yield of almost 70% by simultaneous wet bead milling and enzymatic saccharification of milled wood. Additional data, including the process energy requirements, would be required to compare the cost-effectiveness and scalability of this purely mechanical approach against other pretreatments, including the one described here.

**Fig. 5:**
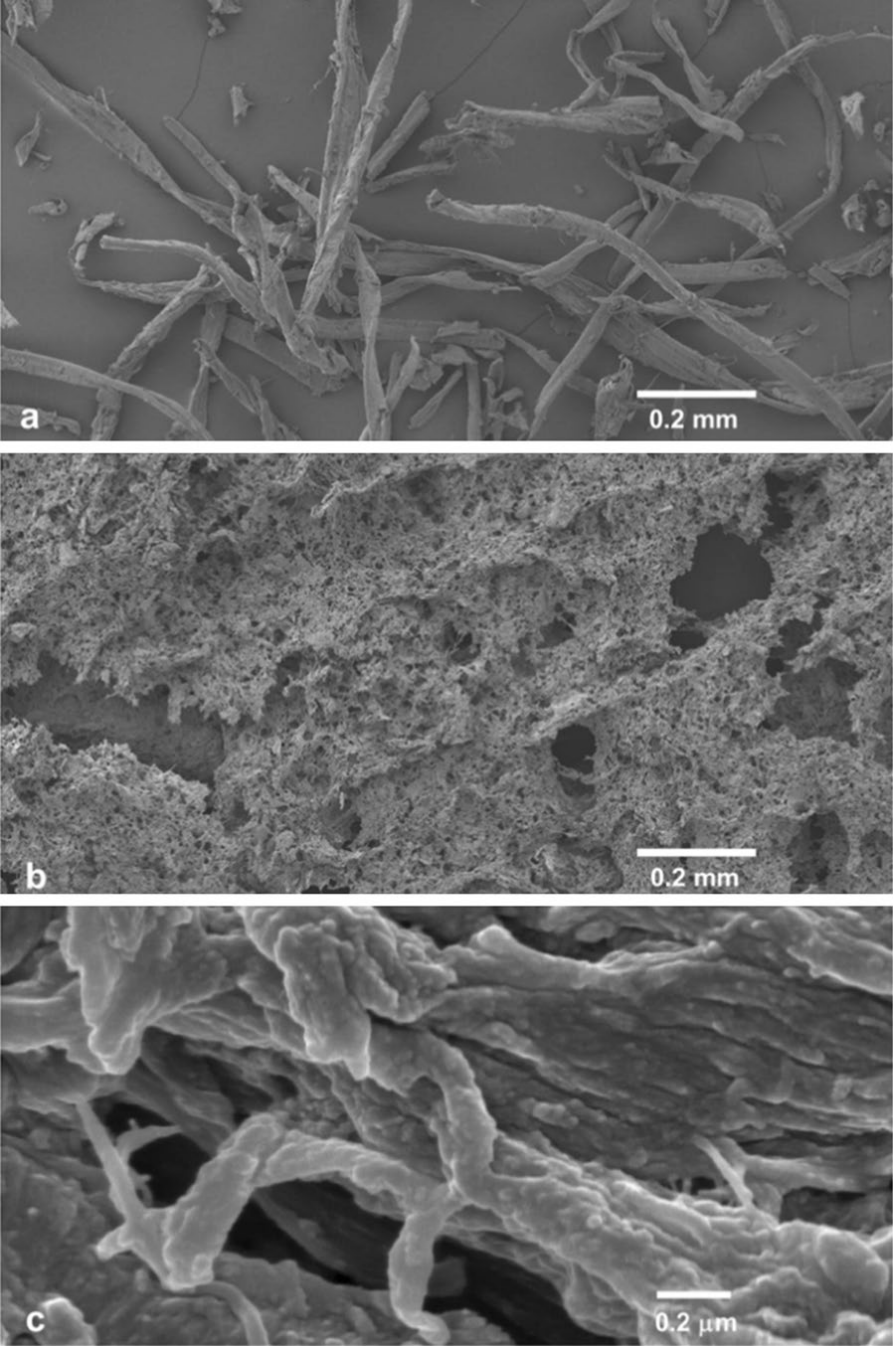
Field emission scanning electron microscope images of **a** fibre produced following steaming for 72 min; **b** the same fibre following ball-milling for 60 min in the ceramic vibratory ball-mill and freeze-drying; and **c** high-magnification image of the ball-milled fibre showing the delaminated fibre wall with a loosened fibrillar texture

Analysis of the ball-milled fibres by field emission scanning electron microscopy revealed extensive disruption of the fibres after ball-milling to produce much finer cell wall fragments (Fig. 5). The surfaces of these fine fragments also show extensive delamination of the fibre wall with a loosened fibrillar structure. This indicates that ball-milling delaminates the fibre wall, loosens the ultrastructure of the wall fragments and possibly removes some of the matrix material from the fibre surfaces, resulting in greatly increased surface area [39]. This increased surface area on both the outside and inside of the wall fragments explains the enhanced digestibility from ball-milling, as the accessibility of the cellulose to the enzymes is a well-known determinant of digestibility, e.g. Ref. [57].

The benefits of wet ball-milling have been observed using a number of different types of ball-mill, including both steel and ceramic vibratory mills, a 105 L ceramic tumbling ball-mill, a vertical stirred ball-mill and a vibratory rod mill (data not shown). While low or high consistency refining or treatment in a SupermassColloider [23] did enhance the digestibility of the fibre, all were considerably less effective than wet ball-milling when compared at the same energy level (data not shown).

Wet ball-milling using ceramic mills affords a substrate containing some ash (≤6%), as a result of a loss of material from the ceramic balls during milling. The ash content varies depending on the substrate, ceramic ball-milling device used and milling time. All subsequent saccharification results have been corrected for ash in the ball-milled material, as ceramic mills are unlikely to be used on a commercial scale.

The energy required for ball-milling is critical to the overall process economics, but is difficult to confidently estimate by extrapolation from laboratory-scale measurements. The energy of ball-milling of minerals has been reported to decrease as the scale rises as described in Eq.  1 [58]:

1 $$E_{2} = E_{1} \left( {V_{2} /V_{1} } \right)^{0.2}$$

where *E* is the energy required per tonne substrate, *V* is the capacity of the ball-mill and the subscripts 1 and 2 refer to the laboratory and industrial equipment, respectively. With our 105 L ceramic tumbling ball-mill processing, a slurry containing 1 kg OD fibre in water at a solid content of 4.8% requires 180 min for acceptable digestion and consumes 1960 kWh/ODT fibre. For scale-up to a ball-mill capable of processing 100 m^3^ of slurry, Eq.  1 predicts that the energy consumption would decrease to 344 kWh/ODT fibre (1.2 GJ/ODT fibre). Further confirmation of this value is required, particularly as it is not yet clear how valid Eq.  1 is for the wet ball-milling of wood fibres. A recent study by Kaufman et al. [59] on the mechanochemical depolymerisation of dry acid-impregnated lignocellulosic materials in a ball-mill reported that the energy consumption for their process significantly decreases as the scale increases. Extrapolating their experimental results to a 100 m^3^ scale predicts an energy consumption even lower than we calculated using Eq. 1.

The energy used during ball-milling needs to be considered in terms of the overall energy used during pretreatment. Table 2 compares the pretreatment energy for this process with steam explosion [60] and the SPORL (Sulphite pretreatment to overcome recalcitrance of lignocellulose) process [61], two other pretreatments proposed for softwoods. The thermal inputs here were determined via thermodynamic calculations using the pretreatment temperature and solids content and assuming 100% efficiency of heat transfer. In practice, the efficiencies will be considerably less than this, but will be offset by recovery of a portion of the thermal energy [60]. While industrially relevant comparisons are not possible here, what Table 2 shows is that size reduction of the steamed chips constitutes approximately half the pretreatment energy for our process and that the higher energy used for size reduction in our process is balanced by a lower thermal energy requirement, with the result that the total energy input lies within the range of the other two processes. The lower thermal energy requirement in our process is a consequence of both the lower pretreatment temperature and the high solids loading, both factors critical to the thermal efficiency [62]. While total pretreatment energies are similar, supplying an increasing proportion of this energy as electricity will normally increase pretreatment energy costs, as electricity is generally more costly than steam on a GJ basis.

**Table 2:** Calculated energy inputs during pretreatment of softwoods

	This process	Steam explosion [60]	SPORL [61]
Wood	Radiata pine	Spruce	Lodgepole pine
Thermal treatment			
T, °C	173	215	180
Solids content, %	40	40	25
Pretreated chip yield, %	85		61
Energy, GJ/ODT wood			
Chipping	0.18	0.18	0.18
Steaming	1.21	1.58	2.33
Disc refining	0.37	–	0.76
Ball-milling	1.05	–	–
Total	2.81	1.76	3.27

The chipping energy was taken from Ref. [62] and the attrition energies are corrected for solids yields following steaming

### Saccharification

**Fig. 6:**
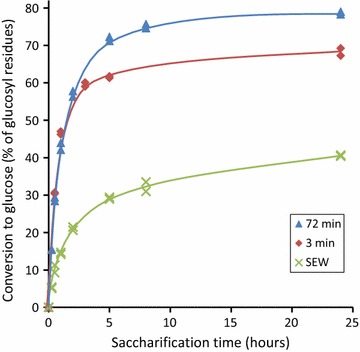
Digestibilities of vibratory ball-milled fibres and reference SEW. Digestibility determined using Celluclast 1.5L (20 FPU/OD g substrate) supplemented with β-glucosidase (Novozyme 188, 25 CBU/OD g substrate). The ball-milling time was 60 min

The extensive delamination and opening up of the fibre wall occurring on ball-milling means the initial rate of hydrolysis of the ball-milled substrate is high, with ca. 90% of the final hydrolysis of glucosyl residues occurring within 5 h (Fig. 6). This is much more rapid than for the reference steam-exploded wood prepared from the same *P. radiata* wood.

**Fig. 7:**
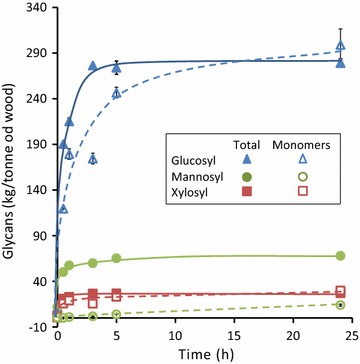
Levels of monomers and total neutral sugar residues solubilised during saccharification of the hemicellulose-rich fibre produced after steaming for 3 min and wet ball-milling for 60 min. Digestibility determined using Celluclast 1.5L (20 FPU/OD g substrate) supplemented with β-glucosidase (Novozyme 188, 25 CBU/OD g substrate)

Both the main hemicelluloses in the substrate, i.e. the GGM and xylan, were rapidly solubilised during enzymatic saccharification. Figure 7 shows that both the mannosyl and xylosyl structural units in the ball-milled substrate were rapidly solubilised during enzymatic saccharification at rates indistinguishable from that of the glucosyl units.

While the solubilised glucosyl and xylosyl units were completely converted to monomers after 24 h enzyme treatment, conversion of the solubilised mannosyl units to mannose (and galactosyl units to galactose, data not shown) was <30% complete (Fig. 7). Our results suggest that the Celluclast/Novo 188 cocktail contains some β-mannanase activity needed to convert the GGM to soluble oligosaccharides, but lacks sufficient β-mannosidase or α-galactosidase activity to completely hydrolyse these oligomers to mannose and galactose. It has been reported that some non-specific cellulases, particularly endoglucanases, have significant β-mannanase side activities towards mannans [63].

### Impact of new cocktails and PEG

**Fig. 8:**
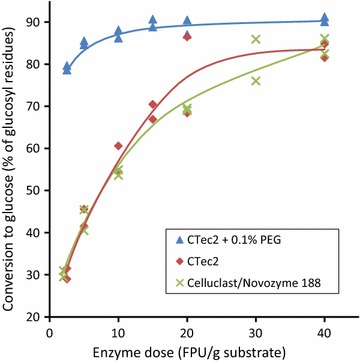
Impact of new cocktail and PEG on enzymatic digestibility. The material produced after steaming for 72 min was attrited in the tumbling ball-mill for 300 min (Cellic CTec2 trials), or vibratory ball-milled for 60 min (Celluclast 1.5L/Novozyme 188 experiments). Trials showed that vibratory ball-milling of this pulp for 60 min and tumble ball-milling for 300 min gave substrates of equivalent digestibilities

With the ball-milled softwood substrates, replacing the Celluclast/Novozyme 188 cocktail with an equivalent dose of the newer Cellic CTec2 cocktail had no major effect on the final extent of conversion (Fig. 8). However, addition of 0.1% w/w PEG (polyethylene glycol 4000) during saccharification greatly improved the effectiveness of saccharification of our substrate using this newer cocktail, particularly at lower enzyme doses. PEG and other additives are believed to be effective because the PEG interacts with the lignin to reduce non-productive binding of enzymes with the lignocellulosic substrates [64–66].

### Overall sugar yields

**Fig. 9:**
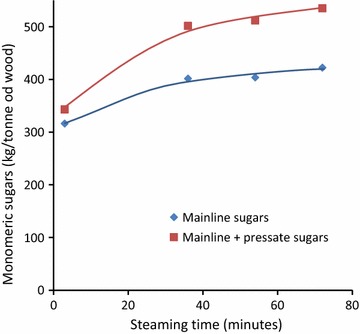
Total monomeric sugar yields (as free sugars) per tonne of input wood as a function of steaming time from pilot scale trials. The refined pulps were vibratory ball-milled (60 min) and digested using Cellic CTec2 (5 FPU/OD g substrate) in the presence of 0.1% PEG. Monomeric sugar yields from the pressates were calculated from the pressate composition assuming the conversion efficiency shown in Fig. 3. Data for steaming times of 36 and 54 min to be reported separately

Increasing the time of steaming in the pilot plant increases the overall yield of sugars per tonne of input wood (Fig. 9). This can be attributed to two factors. Firstly, the digestibility of the pulps after a standard 60-min ball-milling increases as the steaming time is increased (c.f. Fig. 4), more than compensating for the lower pulp yield following steaming. Secondly, hydrolysis of the GGMs increases with increasing steaming time, meaning they are more soluble in the aqueous phase and are separated into the pressate where they can be hydrolysed by acid (c.f. Table 1). In this way, the GGMs are then more efficiently converted to monomeric sugars than would be the case if they remained in the fibre and were only incompletely hydrolysed in the enzymatic saccharification stage.

Total monomeric sugar yields of up to 76% of theoretical are obtained in the process, rising to 83% if the soluble oligomers are included. The latter are mainly as mannosyl residues remaining in the sugar syrup after enzymatic saccharification. This yield is comparable to reported total sugar yields from a range of other acid-catalysed processes for softwoods [3] and recent yields of up to 86% from loblolly pine by bisulfite pulping [34] and 84% from lodgepole pine by the SPORL process [67]. There is also an opportunity to further increase the overall yield by applying higher enzyme doses (c.f. Fig. 8), but this would need to be balanced against the additional cost of the enzyme.

### Fermentation to ethanol

**Fig. 10:**
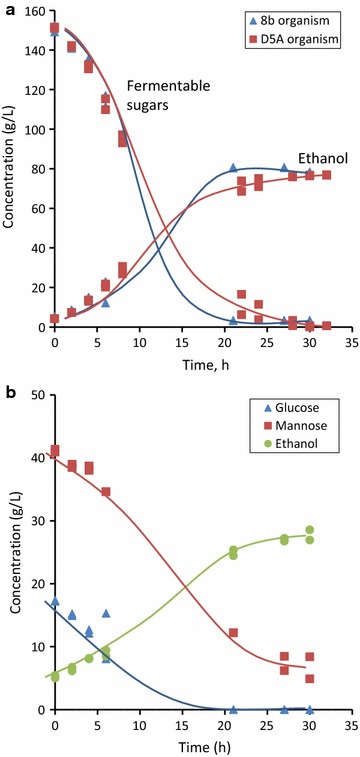
Fermentation of freeze-dried sugar syrups to ethanol. **a** Reconstituted mainline sugar syrup at an initial concentration of 150 g/L of fermentable sugars using both *S. cerevisiae* strain D5A and *Z. mobilis* strain 8b, **b** hydrolysed pressate syrup using *S. cerevisiae* strain D5A reconstituted at an initial concentration of 58.4 g/L fermentable sugars. Fermentable sugars are glucose and mannose for the D5A organism and glucose and xylose for the 8b organism. Results of duplicate experimental determinations shown

Tests undertaken at National Renewable Energy Laboratory showed that freeze-dried samples of both the mainline sugars and hydrolysed pressate sugar syrup produced from chips steamed for 72 min could be readily fermented to ethanol. Fermentation of the mainline glucose-rich syrup at a concentration of 150 g/L sugars using *Saccharomyces cerevisiae* strain D5A (which metabolises both glucose and mannose) and the glucose–xylose co-fermenting bacterium *Zymomonas mobilis* strain 8b gave high ethanol yields, >90% based on fermentable sugars (Fig. 10a). Glucose was completely consumed in both cases. One advantage of our process is that detoxification of this mainline sugar syrup is not required prior to fermentation, consistent with only low levels of fermentation inhibitors produced in the mild thermomechanical pretreatment.

Fermentation of the mannose-rich pressate syrup at a concentration of 58 g/L fermentable sugars using *S. cerevisiae* D5A gave ethanol in a yield of 76% based on the level of fermentable sugars in the pressate (Fig. 10b). Mannose was 87% utilised. The lower sugar utilisation and yields can likely be attributed to partial inhibition by the higher level of acetate in this pressate syrup (see above). In a parallel fermentation using pure mannose as a substrate, the mannose was completely consumed.

## Conclusions

Our process provides an efficient process for the enzymatic conversion of radiata pine, and potentially other softwoods, into a sugar syrup suitable for conversion into fuels and chemicals. The mild thermal pretreatment coupled with wet ball-milling produces only low levels of fermentation inhibitors, meaning that the resulting sugars can be easily fermented to ethanol. In addition, the lignin from the process remains comparatively unmodified, providing an opportunity for conversion into saleable co-products. Furthermore, the process uses standard equipment that is largely proven at commercial scale, reducing risks during commercialisation.

## Methods

### Materials

Enzymes were obtained from Novozymes A/S (Bagsvaerd, Denmark). Filter paper activity units (FPU) were determined according to the IUPAC method and the β-glucosidase activity using *p*-nitrophenyl-β-glucopyranoside as a substrate [68]. Fresh radiata pine (*P. radiata*) wood chips, as produced for use in pulp mills, were obtained from a local sawmill. All other chemicals, including PEG-4000 (MW 4000), were purchased from Sigma-Aldrich (Milwaukee, USA) and used as received.

### Thermomechanical pretreatment

The fibre and pressate samples prepared via trials were performed in the Scion fibre processing pilot plant [40]. This plant operates in a fully continuous mode, with each trial processing approximately 2 m^3^ of chips (~350 OD kg) producing fibre at a flow rate of approximately 17 OD kg/min. Thus, fresh chips were steamed at 80 °C for 5 min in a 2.5 m^3^ steaming vessel and then transferred via a chip compression screw into the 3 m^3^ pressurised steaming vessel maintained at 173 °C (750 kPa) by direct steam injection for 3 or 72 min. The steamed chips were then fed via another chip compression screw (3:1 compression ratio) into the single disc pressurised (650 kPa) refiner (Jylhavaara SD 52/36, 900 mm, 1250 kW) and refined using approximately 300 kWh/ODT energy. The oven dry content of the refined substrates was 50–60%.

The pressates from the two chip compression screw were collected as a single bulked sample for each run, weighed and their solids contents were determined. Mass balances for each trial were calculated on an OD basis from the mass of pulp and pressate solids collected and assuming a pressate density of 1 kg/L and that the mass of input wood chips equals the mass of outputs collected, i.e. no losses occur or volatile compounds are produced through the process.

To produce the “no steaming” fibre, the chips were passed straight through the plant without any steam being applied and were then refined at 0.5 bar inlet pressure and a nominal energy input of 300 kWh/ODT fibre.

### Preparation of steam-exploded wood

*Pinus radiata* wood chips were steam-exploded following the procedure of Clark and Mackie [29] using the optimum conditions they identified for this species. Briefly, a sample of fresh chips (0.75 OD kg) was impregnated with SO_2_ (3% w/w) and heated with steam in a 3 L steam gun at 215 °C for 3 min before being rapidly discharged into a cyclone. The resulting solid was washed four times with deionised water to obtain a 54% yield of water-insoluble substrate.

### Ball-milling

Vibratory ball-milling was carried out for the required time in 1-L porcelain pots on a Schwingmühle VIBRATOM vibratory ball-mill loaded with two hundred 15-mm-diameter alumina balls (ca 1350 g), never-dried pulp (6 OD g) and sufficient 0.01% w/v aqueous solution of sodium azide to give solids content of 4.8%. The slurry of milled solids was transferred from the pots with the aid of additional water and stored at 4 °C.

Tumble ball-milling was performed in a porcelain-lined 105 L tumbling ball-mill equipped with 20-mm-diameter alumina balls. This was loaded with never-dried pulp (1 OD kg) plus water to bring the solids content to 6% and then sealed and rotated for the required length of time. The slurry of milled solids was removed from the mill and stored at 4 °C after the addition of 0.01% w/v sodium azide (unless required for fermentation trials). For a milling time of 180 min, the energy consumption was 1.96 Wh per OD kg fibre as determined by a Metec DVH3113 energy transducer.

### Pressate hydrolysis

Sulphuric acid (24% w/w, 21 mL) was added to the pressate (479 mL, centrifuged and filtered through a 0.45-µm cellulose acetate filter) and heated at 121°C for 1 h in a large laboratory autoclave. The resulting solution was cooled and adjusted to pH 3 by addition of 33% w/w aqueous ammonia (11.3 mL) to give a hydrolysed pressate (456 mL), which was then frozen, freeze-dried or stabilised with 0.01% sodium azide prior to further analysis.

### Enzymatic hydrolysis

Enzymatic hydrolysis was performed in duplicate on a 5-mL scale at 50 °C using 0.05 M sodium citrate buffer (pH 4.8) containing 0.01% sodium azide at a substrate concentration of 1.5% on a dry basis in 20 mL screw-capped glass tubes. The enzyme cocktails that used either a mixture of Celluclast 1.5L supplemented with β-glucosidase (Novozyme 188 at a ratio of 1 FPU: 1.25 IU β-glucosidase) or Cellic CTec2 were added and the tubes agitated at 180 rpm for the required time in an inclined vibratory shaker. If required, PEG-4000 (0.1% w/v) was also added. The reaction was stopped by plunging the tubes into a boiling water bath for 5 min and then cooling to room temperature. The mixture was then centrifuged at 4000 rpm for 10 min at 25 °C and the concentration of glucose in the supernatant was determined using an YSI-2700 glucose analyser (YSI Incorporated). All results are expressed as anhydroglucose units and are corrected for glucose present in a control treatment carried out as described above, but using denatured enzymes.

### Analyses

The total lignin content was determined on extracted samples as the sum of Klason plus acid-soluble lignins following standard methods (Tappi standard T222 om-88 1988; Tappi standard UM-250 1991) scaled down to analyse 0.25 g wood. Extractives were removed by extraction of the ground samples with dichloromethane in a Soxtec apparatus (Tecator Soxtec System Model HT1043) using a boiling time of 1 h and a rinsing time of 1 h. Monomeric sugars in the filtrates from Klason lignin determinations were analysed by ion chromatography [69] and the results were expressed as the corresponding anhydrosugar units (glucosyl, xylosyl etc.). Carbohydrates in pressates were similarly analysed in duplicate before (monomeric sugars) and after (total sugars) hydrolysis in 4% sulphuric acid at 121 °C in an autoclave for 60 min. All biomass and liquor analyses were performed in duplicate, with either the replicates shown, or the mean and deviation from mean reported.

Ash in solid samples was determined by ashing at 525 °C following Tappi standard T211 om-93.

Furans and volatile fatty acids in the filtered pressates were determined in duplicate by ion chromatography using an Aminex HPX-87H column following the method of Sluiter et al. [70].

Releasable β-ethers in in situ lignin were determined in duplicate by thioacidolysis and analysis of silylated monomeric thioacidolysis products by gas chromatography/mass spectroscopy following the method of Pasco and Suckling [50].

Uncondensed phenylpropane lignins in the in situ fibre lignins were determined in duplicate by nitrobenzene oxidation following the method of Chen [51].

Samples for field emission scanning electron microscopy were washed, centrifuged and freeze-dried. The freeze-dried pellets were split open to reveal an internal surface and a portion of this surface was mounted on a carbon-adhesive tab on an aluminium sample holder and sputter-coated with chromium. Samples were examined at an accelerating voltage of 3–5 kV on a JEOL 6700F instrument.

### Fermentation

Fermentation experiments were performed in duplicate at the National Renewable Energy Laboratory, using both *Zymomonas mobilis* strain 8b (a glucose + xylose co-metabolising strain) and *Saccharomyces cerevisiae* D5A (preferentially glucose metabolising strain). The sugar syrups used were produced from chips steamed for 72 min and large-scale pressate hydrolysis or saccharification of pulp tumble ball-milled for 300 min using Cellic CTec2 (20 FPU/OD g substrate) for 48 h at 50 °C and 5% solids loading. The freeze-dried syrup powders were rehydrated into the appropriate concentrations: 150 g/L fermentable sugars (glucose + xylose for *Z. mobilis* 8b and glucose + mannose for *S. cerevisiae* D5A) for the mainline sugar syrup and 58.4 g/L fermentable sugars for the pressate syrup. Fermentations of the sugar syrup by *Z. mobilis* 8b and the hydrolysed pressate using *S. cerevisiae* D5A were accompanied by pure sugar fermentations as controls with matching sugar concentrations and nutrients (data not shown).

The inoculum was prepared by adding 1.0 mL of thawed cell suspension from a cryovial into 9 mL of fermentation medium. The fermentation medium for *Z. mobilis 8b* was 10 g/L yeast extract and 2 g/L KH_2_PO_4_ supplemented with 100 g/L glucose and 20 g/L xylose. For *S. cerevisiae D5A*, an identical medium was used, except KH_2_PO_4_ was replaced with 20 g/L peptone and xylose was replaced with an equal concentration of mannose. The inoculum was incubated at 33 °C (*Z. mobilis* 8b) or 37 °C (*S. cerevisiae D5A*) for 8 h and then the optical density was measured at 600 nm.

Once the optical density of the inoculum reached 0.01 units, it was transferred to the Biostat Q-Plus fermenter containing 300 mL of corresponding fermentation medium (in the case of *Z. mobilis* 8b, the xylose concentration in the medium was increased to 20 g/L) supplemented with 1 g/L sorbitol as an internal standard. For the *Z. mobilis 8b*, fermentation was performed at 33 °C and 300 rpm with the pH controlled at 5.75 using 4 M KOH. The fermentation continued until the OD_600nm_ reached approximately 10 units (about 17 h). The *S. cerevisiae D5A* fermentation was performed at 37 °C and 300 rpm and pH was controlled at 5.10 using 4 M NaOH. The fermentation continued until the OD_600nm_ reached approximately 15 units (about 18 h). Then cultures were transferred to the sugar syrup or pure sugar solutions at a volume ratio of 1:9 and fermentation continued for 30 h. Both fermentations were performed in duplicate.

Ethanol concentrations were monitored by HPLC using a BioRad HPX-87H organic acid column and sugar concentrations were measured by HPLC using a Shodex SP0810 carbohydrate column [70]. Because mannose and arabinose co-elute using this column, arabinose concentrations were determined by ion chromatography using a Dionex PA1 column [69] and used to calculate the mannose concentrations.

## Abbreviations

CBU: cellobiase activity units
FPU: filter paper activity units
GGM: galactoglucomannan
OD: oven dry
ODT: oven dry metric tonnes
OD_600nm_: optical density at 600 nm
PEG: polyethylene glycol 4000
SO_2_: sulphur dioxide
SEW: Steam exploded wood
SPORL: sulphite pretreatment to overcome recalcitrance of lignocellulose
